# Applicability and clinical utility of the German rivermead post-concussion symptoms questionnaire in proxies of children after traumatic brain injury: an instrument validation study

**DOI:** 10.1186/s12883-024-03587-2

**Published:** 2024-04-19

**Authors:** Fabian Bockhop, Sven Greving, Marina Zeldovich, Ugne Krenz, Katrin Cunitz, Dagmar Timmermann, Matthias Kieslich, Nada Andelic, Anna Buchheim, Inga K. Koerte, Maike Roediger, Knut Brockmann, Michaela V. Bonfert, Steffen Berweck, Michael Lendt, Michael Staebler, Nicole von Steinbuechel

**Affiliations:** 1 University Medical Center Göttingen, Göttingen, Germany; 2 Department of Paediatric Neurology, Goethe-University Frankfurt/Main, Frankfurt/Main, Germany; 3 Research Centre for Habilitation and Rehabilitation Models and Services (CHARM), Department of Health and Society, University of Oslo, Oslo, Norway; 4 Department of Physical Medicine and Rehabilitation, Oslo University Hospital, Oslo, Norway; 5 Institute of Psychology, Faculty of Psychology and Sport Science, University of Innsbruck, Innsbruck, Austria; 6 Department of Child and Adolescent Psychiatry, Psychosomatics, and Psychotherapy, Ludwig‑Maximilians‑Universität München, Munich, Germany; 7 Department of Pediatric Intensive Care Medicine and Neonatology, University Hospital Münster, Münster, Germany; 8 Department of Pediatrics and Adolescent Medicine, University Medical Center Göttingen, Göttingen, Germany; 9 Department of Pediatric Neurology and Developmental Medicine, LMU Center for Development and Children With Medical Complexity, Ludwig‑Maximilians‑Universität München, Munich, Germany; 10 Specialist Center for Paediatric Neurology, Neurorehabilitation and Epileptology, Schoen Klinik, Vogtareuth, Germany; 11 Neuropediatrics, St. Mauritius Therapeutic Clinic, Meerbusch, Germany; 12 Neurological Rehabilitation Center for Children, Adolescents and Young Adults, Hegau-Jugendwerk GmbH, Gailingen am Hochrhein, Germany; 13 Institute of Psychology, University Innsbruck, Innsbruck, Austria; 14 Faculty of Psychotherapy Science, Sigmund Freud University Vienna, Vienna, Austria; 15 Department of Psychosomatic Medicine and Psychotherapy, Division of Medical Psychology and Medical Sociology, University Medical Center Göttingen, Göttingen, Germany; 16 Psychiatry Neuroimaging Laboratory, Department of Psychiatry, Mass General Brigham, Bosten, USA

**Keywords:** Rivermead post-concussion symptoms Questionnaire, Children, Traumatic brain injury, Proxy rating, Psychometric properties

## Abstract

**Background:**

The German Rivermead Post-Concussion Symptoms Questionnaire (RPQ) can be used to assess post-concussion symptoms (PCS) after traumatic brain injury (TBI) in adults, adolescents, and children.

**Methods:**

In this study, we examined the psychometric properties of the German RPQ proxy version (*N* = 146) for children (8—12 years) after TBI at the item, total and scale score level. Construct validity was analyzed using rank correlations with the proxy-assessed Post-Concussion Symptoms Inventory (PCSI-P), the Patient Health Questionnaire 9 (PHQ-9), and the Generalized Anxiety Disorder Scale 7 (GAD-7). Furthermore, sensitivity testing was performed concerning subjects’ sociodemographic and injury-related characteristics. Differential item functioning (DIF) was analyzed to assess the comparability of RPQ proxy ratings for children with those for adolescents.

**Results:**

Good internal consistency was demonstrated regarding Cronbach’s α (0.81—0.90) and McDonald’s ω (0.84—0.92). The factorial validity of a three-factor model was superior to the original one-factor model. Proxy ratings of the RPQ total and scale scores were strongly correlated with the PCSI-P (ϱ = 0.50—0.69), as well as moderately to strongly correlated with the PHQ-9 (ϱ = 0.49—0.65) and the GAD-7 (ϱ = 0.44—0.64). The DIF analysis revealed no relevant differences between the child and adolescent proxy versions.

**Conclusions:**

The German RPQ proxy is a psychometrically reliable and valid instrument for assessing PCS in children after TBI. Therefore, RPQ self- and proxy-ratings can be used to assess PCS in childhood as well as along the lifespan of an individual after TBI.

**Supplementary Information:**

The online version contains supplementary material available at 10.1186/s12883-024-03587-2.

## Introduction

Pediatric traumatic brain injury (pTBI) is a significant cause of death and disability in children and adolescents worldwide [1]. Incidence rates of 47 to 280 per 100.000 individuals have been reported for pTBI (mean age 3.2–10.4 years), with rates varying between countries [2]. The most common causes of TBI in children (5—14 years) include falls and sports or recreational accidents [3].

Individuals after pTBI often experience symptoms such as headaches, fatigue, dizziness, and slowed thinking in the acute injury phase, and sleep disturbance, frustration and forgetfulness in the post-acute phase [4]. These symptoms can be collectively referred to as post-concussion symptoms (PCS). While post-concussion-like (PC-like) symptoms are also observed in children and adolescents from general populations [5], somatic PCS in particular are more common [6] and more chronic [7] after pTBI. In the majority of pTBI cases, PCS resolve within the first two weeks [8], but a subgroup of individuals (16%) experiences moderately or highly persistent PCS [9]. The emergence of PCS after pTBI is associated with several sociodemographic, premorbid, familial [10], and cognitive [11] factors. A systematic review [12] found that the risk of persistent PCS was elevated in children and adolescents (2—18 years) who were older, who initially experienced loss of consciousness, headaches, nausea/vomiting or dizziness, or who had premorbid conditions (e.g., previous TBI, learning difficulties, behavioral issues). Validated assessment instruments are essential in order to quantify PCS adequately.

In pediatric settings, outcomes can be assessed either using patient-reported outcome measures (PROM) or their proxy versions, completed by parents or caregivers. PROMs such as the Rivermead Post-Concussion Questionnaire (RPQ) [13], are regularly used in research and in the clinical screening of PCS. The RPQ assesses an individual’s experience of 16 PCS in the physical, cognitive, and behavioral domains (i.e., headaches, dizziness, nausea and/or vomiting, noise sensitivity, sleep disturbance, fatigue, irritability, depression, frustration, forgetfulness and poor memory, poor concentration, slow thinking, blurred vision, light sensitivity, double vision, and restlessness). Previous validation studies of the RPQ in adult populations have reported good to excellent test–retest reliability (r_tt_ = 0.90) [13], split‑half reliability (*r* = 0.82 to *r* = 0.95) and internal consistency (Cronbach’s α: 0.89 to 0.93) [14], and indicated moderate to high convergent validity and good discriminant validity [14]. Excellent psychometric properties have been reported for multiple translations of the RPQ, including the German version [14, 15]. This makes the RPQ particularly suitable for use in international research and practice.

In the field of pTBI, the English RPQ has most frequently been used in samples of concussed adolescent and young adult athletes (14—20 years) [16]. No systematic psychometric validation of the RPQ for pre-teen children has been presented to date. In one of the first studies to focus on children and adolescents (12 ± 3 years), Gagnon and colleagues [17] reported good concurrent validity of the RPQ with regard to clinical group differences between concussed and non-concussed individuals. No further information was provided on other psychometric properties. In a recent study by our group [18], we found evidence for the sensitivity of several outcome measures, including the RPQ, across sociodemographic (i.e., sex, age, education), premorbid (psychological health status), and injury-related (i.e., clinical care pathways, TBI and extracranial injury severity) factors in individuals after TBI. The RPQ was included in the Common Data Elements (CDE) [19] recommendations as a supplementary instrument for adults. However, it has repeatedly been used in pediatric settings [20, 21], calling for further validation.

For assessing PCS following pTBI, the CDE recommendations suggest instead the use of the Post-Concussion Symptom Inventory (PCSI) [22]. This instrument, originally developed in English, is available as a validated self-report and proxy version (PCSI-P) that can be applied to children aged 5—17 years [23]. The PCSI and the RPQ are comparable in terms of item content. It should be noted that separate age-adapted forms of the PCSI are used to assess PCS before and after pTBI, whereas the RPQ can be used to assess both aspects in one version.

Proxy ratings are often used to assess children’s mental health problems after pTBI (e.g., 24), offering an additional perspective on the effects of the injury and subsequent therapeutic interventions. Proxy-assessed versions of the RPQ have been evaluated in pediatric samples (4—17 years) in English [24] and, most recently, specifically in adolescents after pTBI in German [25]. The evaluation of the German RPQ proxy version in pre-adolescent children would be unique in improving the longitudinal assessment of PCS across the lifespan of patients using the same instrument. Proxies tend to report lower rates of impairment with regard to PCS and PC-like symptoms [26], resulting in only moderate parent-child concordance [23]. The relatively low congruence between self and proxy ratings and the position of children as experts on their own subjective health status underline the general importance of self-reported PROMs. However, proxies can provide useful additional information in cases where the children are unable to respond for themselves or where their awareness is too severely impaired for a reliable self-report. A systematic evaluation of the German RPQ proxy for children may consolidate the validity and utility of the RPQ as a tool for assessing PCS after pTBI. Since German is the most widely spoken language in the EU after English, the validation of a German version of the RPQ is relevant for a large number of individuals.

The present study therefore aims to investigate the psychometric properties of the proxy version of the German RPQ for the assessment of PCS in accordance with the COSMIN Taxonomy of Measurement Properties [27]. The age group analyzed was chosen to correspond to the age groups considered in the PCSI instruments (i.e., PCSI-SR5: 5–7 years, PCSI-SR8: 8–12 years, and PCSI-SR13: 13–18 years) [22]. In order to allow analyses to be carried out for comparable age groups, we focused on children aged 8–12 years. Results indicating acceptable psychometric properties would suggest that the RPQ proxy version can be used to reliably and validly assess the presence and severity of PCS after pTBI. A secondary aim is to compare RPQ scores by children’s proxies with the RPQ scores of adolescents’ proxies [25]. We expect no significant differences in response behavior, indicating that RPQ proxy ratings can be applied for children as well as for adolescents, thus underlining the utility of the instrument in a clinical context.

## Materials and methods

### Study population

The current study is part of the Quality of Life after Brain Injury for Children and Adolescents (QOLIBRI-KID/ADO) multicenter project to develop the first TBI-specific health-related quality of life questionnaire, which was conducted at 12 medical centers in Germany from January 2019 to January 2022. The retrospective, clinical convenience sample for this study was recruited in multiple steps. First, lists of patients were obtained from recruiting centers, which contained information on pediatric subjects who had received a diagnosis of TBI (ICD code S06.*) in the past ten years. Next, individuals were invited if they met the following inclusion criteria: age 8—17 years, diagnosis of TBI (three months to ten years prior to study enrollment), availability of information on TBI severity (based on either the Glasgow Coma Scale (GCS) [28] or clinical records), and the ability to comprehend and complete the study assessment. The exclusion criteria were a diagnosis of epilepsy or severe mental illness prior to TBI, very severe polytrauma, and diseases leading to death. Finally, written informed consent was obtained from parents or legal guardians and online or in-person assessments were scheduled at the respective centers.

**Fig. 1:**
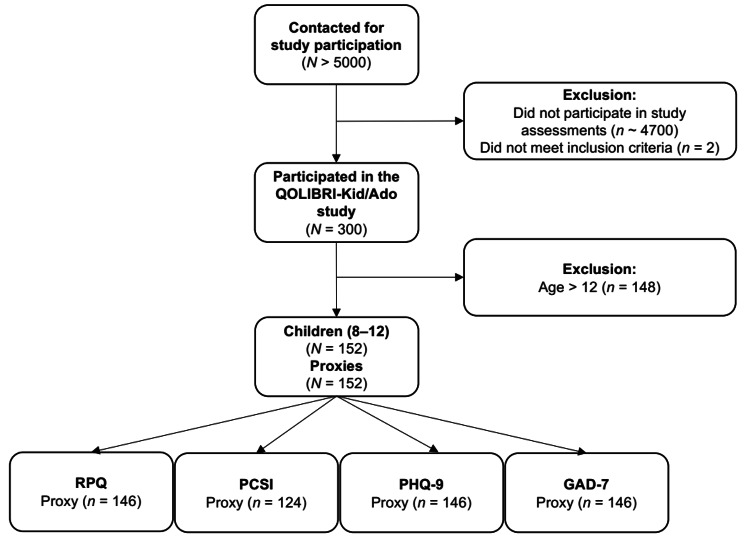
Sample attrition diagram

More than 5000 families were contacted to participate in the QOLIBRI-KID/ADO study. In the end, a total of 300 participants (8—12 years: *n* = 152; 13—17 years: *n* = 148) were interviewed either online (8—12 years: *n* = 39; 13—17 years: *n* = 37) or in person (8—12 years: *n* = 113; 13—17 years: *n* = 111). Although the overall response rate was relatively low (7%), the sample size was appropriate for the main analyses based on a priori sample size estimates (i.e., at least 140 participants and their proxies per age group) [29]. Proxy ratings were obtained from parents and caregivers using paper-pencil questionnaires (8—12 years: *n* = 146; 13—17 years: *n* = 147). The psychometric evaluation of the RPQ in the adolescent subsample can be found elsewhere [25]. The current study focuses on the proxy ratings of the German RPQ obtained from the parents of children after pTBI aged 8—12 years. Based on the findings from adult research using the RPQ [30–32] and knowing that individuals after moderate to severe TBI may also experience similar so-called post-concussion-like symptoms [13, 33], cases across the entire TBI severity spectrum were included in the current study. Figure 1 provides an overview of sample attrition.

### Sociodemographic and injury-related data

Sociodemographic and clinical data were collected either from parent reports or from medical records. The sociodemographic characteristics comprised the gender and age of the children and their proxies.

The clinical information included TBI severity, classified as mild, moderate, or severe; time since injury in years; and the presence of lesions observable on neuroimaging scans (none vs. at least one lesion in CT or MRI). The Kings Outcome Scale for Childhood Head Injury (KOSCHI) [34] was used to classify the functional recovery after pTBI at baseline as 3a = ‘lower severe disability’, 3b = ‘upper severe disability’, 4a = ‘lower moderate disability’, 4b = ‘upper moderate disability’, 5a = ‘good recovery’, and 5b = ‘full recovery’.

### Patient-reported outcome measures (PROMs)

#### Rivermead post-concussion symptoms questionnaire (RPQ)

The RPQ [13] is a PROM that rates the presence and severity of 16 PCS in comparison to the individual’s condition before the TBI on a five-point Likert-type scale (0 = ‘not experienced at all’, 1 = ‘no more of a problem than before’, 2 = ‘a mild problem’, 3 = ‘a moderate problem’, and 4 = ‘severe problem’). The total score is computed as the sum of all individual item ratings above ‘1’ (i.e., higher impairment after compared to before the TBI). It ranges from 0 (no increased impairment) to 64 (most pronounced difficulties) with a clinical screening cut-off at 12 [35]. The present study used the proxy version of the German RPQ, which was developed and validated in adolescents after TBI [24]. The adjustments made to the RPQ for proxy assessment included changing the wording in the instruction from “Do you suffer from…” to “Does your child suffer from…”. In cases of missing values on one to four RPQ items, imputation by prorating the scale mean was applied. Individuals with five or more missing values were excluded from further analyses.

#### Post-concussion symptom inventory (PCSI)

The PCSI [22, 23] can be administered to report the subjective experience of PCS relative to before the TBI. The current study used the proxy form of the PCSI (i.e., PCSI-P) [23] which comprises 21 items assessing PCS as well as the individual’s overall health condition. The PCSI-P is rated on a seven-point Likert-type scale (from 0 = ‘not a problem’ to 6 = ‘severe problem’). Ratings can be summarized in the form of a total score which ranges from 0 to 132, as well as separate cognitive, emotional, somatic, and fatigue scales, with higher values indicating a higher symptom burden. Previous research [23] has administered the English PCSI-P in children and adolescents (5–18 years) and reported good to excellent internal consistency of the total score (Cronbach’s α = 0.94) and its scales (Cronbach’s α: 0.83–0.92), as well as evidence for good convergent validity, good predictive validity, and fair factorial validity. In the present study, only the post version (i.e., assessing symptoms after injury) was used. A detailed description of the translation process of the German PCSI can be found elsewhere [36].

#### Patient health questionnaire 9 (PHQ-9)

The PHQ-9 [37] comprises nine symptoms of major depression. Impairment is rated on a four-point Likert scale (from 0 = ‘not bothered at all’ to 3 = ‘bothered nearly every day’). Total scores range from 0 to 27, with higher values indicating more severe depression symptoms. Values from 1 to 4 represent minimal depression, and scores equal to or greater than 5, 10, or 15 indicate mild, moderate, or severe depression, respectively [37, 38]. A recent study administered the English PHQ-9 proxy for children and adolescents after mild TBI and reported preliminary evidence of good internal consistency (Cronbach’s α: 0.78–0.83) [39]. Moreover, a recent study using a modified version of the PHQ-9 proxy in English-speaking older adults (75.3 ± 7.0 years) reported good internal consistency (Cronbach’s α: 0.83) and excellent test-retest reliability (r_tt_ = 0.92) [40]. The PHQ-9 had previously been translated into German and validated in individuals after TBI [14]. The present study used a proxy-adapted version of the German PHQ-9 which differed from the self-reported version solely regarding the wording in the introductory instructions (i.e., “…how often have you been bothered by…” vs. “…how often has your child been bothered by…”).

#### Generalized anxiety disorder scale 7 (GAD-7)

The GAD-7 [41] assesses seven symptoms associated with generalized anxiety disorder. Impairment is rated on a four-point Likert scale (from 0 = ‘not bothered at all’ to 3 = ‘bothered nearly every day’). Total scores range from 0 to 21, with higher values indicating more severe anxiety symptoms and scores above 5, 10, or 15 representing mild, moderate, or severe impairment, respectively [41]. Previous studies in pTBI samples have focused on the GAD-7 self-report for assessing anxiety, e.g., [42]. With regard to proxy assessments, a recent study used the GAD-7 proxy in a sample of English-speaking older adults (75.3 ± 7.0 years) and reported good internal consistency (Cronbach’s α: 0.87) and test-retest reliability (r_tt_ = 0.74) [40]. The GAD-7 had previously been translated into German and validated in individuals after TBI [14]. The present study used a proxy-adapted version of the German GAD-7 which differed from the self-reported version solely regarding the wording in the introductory instructions (i.e., “…how often have you been bothered by…” vs. “…how often has your child been bothered by…”).

### Statistical analyses

Descriptive analyses were conducted for sociodemographic characteristics, injury-related information and PROM data. Reliability and validity analyses at the item and scale level were carried out for outcome data obtained using the proxy version of the German RPQ.

### Item characteristics

We calculated the absolute (n) and relative frequencies (%) of missing values, means (M), and standard deviations (SD). Moreover, skewness (SK) and kurtosis (KU) were computed, with values between − 2 and + 2 considered acceptable [43]. Proxies’ response behavior was evaluated for each item using the distribution (n and %) of responses. Floor or ceiling effects were considered to exist when more than 15% of the responses were assigned to the lowest or highest response categories, respectively [44]. A Shapiro-Wilk normality test was performed to test the distribution of the total and scale scores for normality.

### Reliability

The reliability of the RPQ was analyzed based on Cronbach’s α and McDonald’s ω. Values from 0.70 to 0.95 (Cronbach’s α) and above 0.80 (McDonald’s ω) indicated good to excellent internal consistency [44]. In addition, changes in Cronbach’s α after omitting individual items were computed. Results that exceed the initial α would indicate a higher consistency of the scale without the item in question. Corrected item-total correlations (CITC) indicated the association between individual items and the scale scores, with *r* ≥ 0.30 considered acceptable [45].

### Factorial validity

The factor structure of the RPQ was originally proposed to be unidimensional [13], however this conceptualization has frequently been challenged [30]. An alternative three-factor model including cognitive, emotional, and somatic scales has been found to have a superior fit in adolescents [25] and adults [30]. We therefore examined the fit of the original one-factor model as well as the three-factor model. For this purpose, a confirmatory factor analysis (CFA) was conducted with a robust weighted least-squares estimator (WLSMV) [46] for ordinal data. In cases of a limited use of the response categories in individual items, the response categories were collapsed to enable robust model estimations. The factor models were considered to have a good fit if the following cut-off criteria were met in the respective indices (see parentheses): Comparative Fit Index (CFI ≥ 0.95), Tucker-Lewis-Index (TLI ≥ 0.95), standardized root mean square residual (SRMR ≤ 0.08), and root mean square error of approximation with 90% confidence interval (RMSEA [CI90%]; mediocre fit at 0.10, excellent fit at 0.05) [47]. Furthermore, the ratio of chi-square to degrees of freedom (*df*) served as a measure of goodness-of-fit and values < 2 indicated a good fit [48]. Scaled statistics are reported for all indices except SRMR. All indices were considered simultaneously based on findings indicating that the cut-offs provided should be treated with caution in model estimations using categorical data [49].

### Validity

The analyses of construct validity were based on the convergence of the RPQ proxy with the PCSI-P. Comparisons with the proxy-rated GAD-7 and the PHQ-9 were used to assess convergent and discriminant validity. We also tested hypotheses regarding emotional symptoms and sociodemographic and clinical indicators.

To account for the non‑normal distribution of the outcome data, correlational analyses were performed between the RPQ proxy and the PCSI‑P total data, as well as scale scores using Spearman’s ϱ. Cohen’s conventions were applied to describe the effect size of the respective correlation coefficients as weak (|0.10| ≤ ϱ < |0.30|), moderate (0.30 ≤ |ϱ| < 0.50), or strong (ϱ ≥ |0.50|) [45]. We expected strong positive correlations between the RPQ proxy and PCSI‑P scores (i.e., ϱ ≥ 0.50) which would indicate that both measures assess the same underlying construct.

Convergent and discriminant validity were also assessed using rank correlations between proxy-assessed RPQ total and scale scores and proxy-rated anxiety (GAD-7) and depression (PHQ-9). Correlations between the RPQ total score and emotional scale against the GAD-7 and PHQ-9 total scores were expected to be positive and at least moderate (i.e., ϱ ≥ 0.30), indicating adequate convergent validity. The discriminant validity of the somatic and cognitive scales with anxiety and depression was considered acceptable for small correlations (ϱ **<** 0.30).

To further investigate construct validity, we tested a number of hypotheses with regard to gender, TBI severity and functional recovery with the RPQ proxy data. We expected higher RPQ values in girls compared to boys [50], in children suffering from moderate-severe TBI as compared to mild TBI [51], and in individuals who had not fully recovered (KOSCHI < 5b) after TBI [52]. With regard to different levels of anxiety and depression (i.e., no or minimal symptom burden: 0–4 vs. at least mild symptom burden: ≥5), higher RPQ values were hypothesized to be associated with more severe emotional distress [53]. Finally, we tested whether the RPQ scores of participants with parent-reported sensory, cognitive, and/or physical post-TBI problems (i.e., problems with taste, hearing, vision, speech and language, learning, extremities and movement, or seizures) differed significantly from those whose parents reported no such health conditions. All hypothesis tests were based on nonparametric Mann–Whitney U-tests for independent data. The effect size in the groups comparisons was estimated using Cliff’s δ with the following cut‑offs: δ < |0.28| (small), 0.28 < |δ| < 0.43 (medium), and δ ≥ |0.43| (large) [54].

### Differential item functioning

We further investigated the RPQ by comparing the response behavior of children’s proxies with that of adolescents’ proxies. Analyses of differential item functioning (DIF) employing logistic ordinal regression (LORDIF) [55] were used to detect meaningful deviations in response behavior. The RPQ proxy data for children was combined with previously reported RPQ proxy data of adolescents [25]. The combined data set was then used to calculate two regression models. In these, individual symptom ratings served as outcome variables alongside the following potential predictors: [1] scale mean, [2] scale mean, age group (children vs. adolescents), age-group/scale-mean interaction. Both models were compared by means of chi-square tests, where deviations were considered meaningful if the differences were statistically significant (*p* < 0.01) as well as if at least a very small effect (i.e., McFadden’s Pseudo R² > 0.05) [56] was detected.

The analyses were conducted in R (version 4.1.0) [57] using the packages lavaan [58], psych [59], and lordif [60]. The significance level was set at 5% for all analyses unless otherwise noted. For multiple comparisons of the RPQ scale scores, the significance level was adjusted using the Bonferroni correction.

## Results

### Sample characteristics

Table 1 displays the characteristics of the sample. The sample consisted mostly of boys (61%) and the average age was 10.63 ± 1.40 years. The majority of children sustained a mild pTBI (70%) two to ten years before study enrollment (82%), had no brain lesions (71%), and had fully recovered (KOSCHI score 5b; 85%) at the time of assessment. The proxies were most often mothers (76%) and had a mean age of 44.62 ± 5.15 years.

**Table 1:** Sociodemographic and injury-related characteristics of the study population

Variable	Group/Values	Children (*n* = 152)	Proxies (*n* = 152)
Gender†	Female	58 (38%)	116 (76%)
	Male	94 (62%)	30 (20%)
	Missing	0 (0%)	6 (4%)
Age	*M* (*SD*)	10.63 (1.40)	44.62 (5.15)
	*Min–Max*	8.00–12.92	32.00–55.00
TBI severity†	Mild	106 (70%)	—
	Moderate	16 (11%)	—
	Severe	30 (20%)	—
Number of suspected lesions†	No lesions	108 (71%)	—
	At least one lesion	43 (28%)	—
	Missing	1 (1%)	—
Years since injury†	< 1	4 (3%)	—
	1–<2	24 (16%)	—
	2–<4	45 (30%)	—
	4–10	79 (52%)	—
KOSCHI Score†	3a (lower severe disability)	0 (0%)	—
	3b	1 (1%)	—
	4a	3 (2%)	—
	4b	4 (3%)	—
	5a	15 (10%)	—
	5b (full recovery)	129 (85%)	—

† For categorical variables, absolute (*n*) and relative (%) frequencies are reported.

TBI = traumatic brain injury, KOSCHI = Kings Outcome Scale for Childhood Head Injury, *M* = mean, *SD* = standard deviation, *Min* = minimum, *Max* = maximum. Percentages may not sum up to 100% due to rounding.

Overall, the total scores of the RPQ proxy reports (as well as in the PCSI-P) indicated the experience of mild PCS. More specifically, the average RPQ scale scores ranged between 1.54 and 2.75 and the average PCSI-P scale scores between 1.17 and 3.35. According to the rating scales, these values correspond to the experience of moderate problems in both the RPQ and the PCSI-P. In addition, the average proxy scores on the PHQ-9 (*M* = 3.93, *SD* = 3.36) and the GAD-7 (*M* = 3.56, *SD* = 3.08) indicated mild impairment. Most proxies reported no to minimal depression (67%) and anxiety (66%), respectively. The Shapiro-Wilk tests revealed significantly non-normal distributions in the total score and all scale scores. For an overview of the mean scores on the psychopathological instruments observed in the current study, see Table 2.

**Table 2:** Descriptive statistics of the total and scales scores of the proxy-rated psychopathological instruments

	Scale	n	M	SD	Mdn	W
RPQ proxy	Cognitive	146	1.54	2.80	0	**0.62**
	Somatic	146	2.75	4.59	0	**0.65**
	Emotional	146	2.25	3.43	0	**0.70**
	Total score	146	6.54	9.41	2	**0.73**
PCSI-P	Cognitive	124	2.81	4.41	1	**0.68**
	Physical	124	3.35	5.27	1	**0.68**
	Emotional	124	3.15	3.92	2	**0.79**
	Fatigue	123	1.17	2.76	0	**0.50**
	Total score	123	10.54	14.55	5	**0.73**
PHQ-9 proxy	Total score	146	3.93	3.36	3	**0.89**
GAD-7 proxy	Total score	146	3.56	3.08	3	**0.90**

*n* = number of observations, *M* = mean, *SD* = standard deviation, *Mdn* = median, *W* = Shapiro–Wilk normality test statistic, RPQ = Rivermead Post‑Concussion Symptoms Questionnaire, PCSI-P = Post‑Concussion Symptom Inventory Proxy Version, GAD‑7 = Generalized Anxiety Disorder Scale 7, PHQ‑9 = Patient Health Questionnaire 9. Values in **bold** indicate significant deviation from normality (*p* < 0.001).

### Item characteristics

The RPQ items averaged *M* = 0.61, *SD* = 0.92, *SK* = 1.93, *KU* = 4.17 with less than 5% missing values. The items displayed a right-skewed distribution with all items yielding floor (*M* = 61%) rather than ceiling (*M* = 2%) effects (see Additional file 1, Table S1).

### Reliability

The internal consistency of the RPQ total score as well as all three scales was good to excellent according to Cronbach’s α and McDonald’s ω, with values ranging from 0.81 (Somatic scale) to 0.90 (Total score) and 0.84 (Somatic scale) to 0.92 (Total score), respectively. No item was found to increase a scale’s α when it was omitted. CITCs indicated at least moderate correlations in the total score as well as across all scales (see Table 3).

**Table 3:** Reliability coefficients of the RPQ proxy version

RPQ Scale	Cronbach’s α	α when item omitted	McDonald’s ω	CITC Range	Correlations		
					(1)	(2)	(3)
(1) Cognitive	**0.87**	0.80–0.84	**0.88**	0.79–0.83	1	–	–
(2) Somatic	**0.81**	0.76–0.80	**0.84**	0.46–0.73	**0.60**	1	–
(3) Emotional	**0.83**	0.72–0.83	**0.86**	0.60–0.89	**0.67**	**0.68**	1
(4) Total	**0.90**	0.89–0.90	**0.92**	0.36–0.78	**0.77**	**0.89**	**0.85**

CITC = Corrected Item-Total Correlation. Values in **bold** indicate at least satisfactory Cronbach’s α (i.e., α ≥ 0.70) and McDonald’s ω (i.e., ω ≥ 0.80) or at least moderate Spearman correlation coefficients (i.e., *r* ≥ 0.30)

### Factorial validity

A preliminary inspection of the data revealed that proxies rarely used response category 4 (‘severe problem), particularly for the item ‘Double Vision’. Consequently, the response categories 3 (‘moderate problem’) and 4 (‘severe problem’) were collapsed for the subsequent analyses. The CFA showed a better fit for the three-factor model compared with the one-factor model, as indicated by more fit indices being above the respective proposed cut-off values (Tables 4 – bold entries). Statistical model comparisons indicated a better fit of the three-factor model for the given data (Tables 4 – right part ‘Model comparison’). Consequently, all further analyses were conducted for the conventional one-factor model as well as the three-factor model.

**Table 4:** Fit indices and model comparisons of the one- and three-factor models for the RPQ proxy

Confirmatory Factor Analyses										Model Comparison	
Model	*χ*2	*df*	*χ*2/*df*	*p*	CFI	TLI	RMSEA	CI90%	SRMR	∆*χ*2 (∆*df*)	*p*
One-factor	225.12	104	2.16	*< 0.001*	**0.96**	**0.95**	0.09	[0.07, 0.11]	0.12	–	–
Three-factor	149.30	101	**1.48**	*0.001*	**0.98**	**0.98**	**0.06**	[**0.04, 0.08**]	0.10	51.44 [3]	*< 0.001*

*χ*2 = scaled chi-square value, *df* = scaled degree of freedom, *χ*2/*df* = ratio (cut-off: ≤ 2), *p* = scaled *p*-value, CFI = scaled Comparative Fit Index (cut-off:  ≥ 0.95), TLI = scaled Tucker-Lewis Index (cut-off: ≥ 0.95), RMSEA = scaled root mean square error of approximation (cut-off: ≤ 0.08) with 90% confidence interval (CI), SRMR = standardized root mean square (cut-off: ≤ 0.08), ∆*χ*2 = chi-square value of the difference test, ∆*df* = degrees of freedom of the difference test. Values in **bold** indicate at least satisfactory model fit according to the respective cut-offs, values in italics are significant at 5%.

### Validity

Table 5 shows the correlations of the RPQ total and scale scores with (a) the PCSI-P total and scale scores, (b) the proxy-assessed PHQ-9 total score, and (c) the proxy-assessed GAD-7 total score. As expected, the RPQ and PCSI total scores and the corresponding scale scores displayed strong positive correlations (i.e., ϱ ≥ 0.50). The PCSI-P fatigue scale was moderately correlated with the RPQ cognitive (ϱ = 0.38) and emotional (ϱ = 0.37) scales, and strongly correlated with the RPQ somatic scale (ϱ = 0.52) and the RPQ total score (ϱ = 0.51).

The correlations of the RPQ total score with the PHQ-9 and the GAD-7 total scores were strong and positive (i.e., ϱ ≥ 0.50). In particular, the RPQ emotional scale was highly correlated with the PHQ total score (ϱ = 0.60) and the GAD-7 total score (ϱ = 0.64). On the other hand, the RPQ cognitive scale was only moderately correlated with the PHQ-9 (ϱ = 0.49) and GAD-7 (ϱ = 0.46) total scores. Interestingly, whereas the RPQ somatic scale was strongly correlated with the PHQ-9 total score (ϱ = 0.57), its association with the GAD-7 total score was moderate (ϱ = 0.44).

**Table 5:** Spearman correlations between the RPQ proxy with the PCSI-P, and the PHQ-9 and GAD-7 proxies

	PCSI-P						
**RPQ Proxy**	Physical	Emotional	Cognitive	Fatigue	Total	**PHQ-9 Proxy**	**GAD-7 Proxy**
Cognitive	**0.52**	**0.53**	**0.65**	0.38	**0.60**	0.49	0.46
Emotional	**0.50**	**0.59**	**0.54**	0.37	**0.57**	**0.60**	**0.64**
Somatic	**0.64**	**0.55**	**0.54**	**0.52**	**0.64**	**0.57**	0.44
Total	**0.62**	**0.62**	**0.64**	**0.51**	**0.69**	**0.65**	**0.57**

RPQ = Rivermead Post‑Concussion Symptoms Questionnaire, PCSI-P = Post‑Concussion Symptom Inventory Proxy Version, PHQ‑9 = Patient Health Questionnaire 9, GAD‑7 = Generalized Anxiety Disorder Scale 7. The PCSI-P total score represents the aggregate of the Physical, Emotional, Cognitive, and Fatigue scales. **Bold** values indicate high correlation coefficients (i.e., ϱ ≥ 0.50).

Group comparisons showed that higher RPQ total and scale scores were associated with more severe TBI, incomplete recovery (i.e., KOSCHI score < 5b), presence of post-TBI sensory, cognitive, and/or physical problems, and the experience of pronounced symptoms of depression and anxiety. No significant differences in the RPQ total and scale scores were observed between boys and girls (see Additional file 1, Table S2)

### Differential item functioning

Differences in the response behavior of children’s proxies and adolescents’ proxies in the RPQ were only observed for one item (‘Forgetfulness, Poor Memory’) as indicated by significant differences between regression models (*p* = 0.007). However, this observed difference did not meet the criterion for an at least very small effect (McFadden *R*^*2*^ = 0.013) and was thus not considered a practically relevant difference (see Additional file 1, Table S3).

## Discussion

The current study focused on evaluating the psychometric properties of the proxy version of the German RPQ for children aged 8–12 years after pTBI. In addition, we compared the RPQ ratings from children’s proxy assessments with those of adolescents’ proxy assessments in order to examine whether the RPQ proxy can be used longitudinally.

We found evidence for good to excellent psychometric properties of the German RPQ proxy as a reliable and valid assessment instrument for PCS after pTBI. Our results underline the sensitivity and clinical utility of the RPQ in identifying differences between individuals with respect to TBI severity and functional recovery status, as well as symptoms of depression and anxiety. Furthermore, the RPQ proxy is a valid tool for the assessment of PCS in children, where needed, just as in adolescents [25]. Summarizing the current findings, together with previous research, we can conclude that RPQ self-reports and proxy ratings can be used as measures of PCS in TBI-affected individuals of various ages and throughout the patient’s life, beginning in childhood (≥ 8 years), through adolescence [25], up to adulthood [13, 14].

Overall, the study population experienced mild PCS, as indicated by RPQ proxy and PCSI-P ratings. RPQ proxy ratings were substantially skewed to the right for all items, with pronounced floor effects. The RPQ total score, as well as the cognitive, emotional, and somatic scales, displayed good to excellent internal consistency. In terms of the item-level analyses, we found high consistencies across the total score and scales as indicated by the CITC. The value for one item (Blurred Vision) was close to the cut-off. Interestingly, previous factor analytic research has found evidence for the presence of a ‘vision-related’ factor in self-reported RPQ data [61]. When the item ‘Blurred Vision’ is combined with related symptoms (e.g., ‘Double Vision’, ‘Light Sensitivity’), evidence for the ‘vision-related’ factor is most pronounced in adults after mild TBI [62] and in the acute phases after TBI [63]. Most children had experienced a pTBI several years before study enrollment. The experience of ‘Blurred Vision’ in pediatric samples should therefore be further investigated, particularly in relation to a ‘vision-related’ factor and its relevance in proxy ratings.

The CFA results for the RPQ proxy data indicated an acceptable fit for the conventional one-factor structure and overall a superior fit for the previously proposed alternative three-factor structure. This finding highlights the importance of differentiating between cognitive, somatic, and emotional PCS, also in proxy ratings after pTBI. However, while most fit indices pointed towards a good to excellent fit for the three-factor RPQ model, the SRMR value failed to meet its cut-off criterion. Previous research has found evidence suggesting that analyses of data with high measurement quality (i.e. high item loadings) paradoxically tend to produce inflated SRMR values [64]. The current study focused on high quality data obtained from a sample with an adequate, albeit relatively small sample size (*N* = 152 proxies) for this type of analysis. Since indices such as the SRMR and RMSEA do not require more stringent cut-off values with increasing sample size [65], replication analyses on the factorial validity of RPQ proxy ratings in larger data sets would further improve the evidence provided by the current study.

We found a strong correlation between proxy-reported PCS and depression and anxiety. Particularly strong positive associations were found between the RPQ emotional scale and the PHQ-9, as well as the GAD‑7 total scores. It should be noted, however, that parent ratings do not always correspond well with clinical interviews on depression and anxiety in children and adolescents after mild TBI [66]. While proxy ratings are commonly used to assess children’s mental health status and neuropsychiatric outcomes after pTBI, parent-child agreement for PCS are modest, with children generally reporting more severe symptoms [39]. In fact, research has suggested that parents’ proxy ratings of persistent PCS after pTBI were more strongly associated with parental stress than with the severity of the children’s injuries [67]. Since RPQ self-report assessments were found to have excellent properties in adult [14, 15] and adolescent [25] populations, the use of RPQ self-reports with age-appropriate item wordings for younger children should be preferred. Overall, proxy versions of the RPQ and related PROMs may be used as surrogates for self-reports or as an additional indicator for clinical treatment of neuropsychiatric outcomes after pTBI. Parents’ own well-being should also be considered when using proxy ratings.

As investigated previously [25], analyses of DIF between RPQ proxy ratings for children and adolescents have revealed a significant but minimal difference for one item (i.e., ‘Forgetfulness, Poor Memory’). More specifically, proxies of adolescents indicated a more severe impairment associated with this item than proxies of children. However, due to its minimal effect, this difference was judged to be of no practical relevance. Overall, we can conclude that the RPQ proxy can be administered for children just as for adolescents and that the resulting RPQ scores are comparable.

Finally, group comparisons showed that, as expected, differences in RPQ proxy ratings could be observed with regard to TBI severity [51], recovery status [52] and the presence of post-TBI sensory [68], cognitive [69], or physical problems [70], as well as the experience of pronounced depression and anxiety [53]. In contrast to previous findings in the literature [50], no significant difference was observed with regard to gender. While previous research has reported differences in RPQ self-reports in adults after mild TBI, these differences were most prominent in females of child-bearing age [71]. Gender differences in outcomes after pTBI seem to be driven by multiple biological factors, including sex hormones, steroid hormones and cellular activity, e.g. [72]. While biological sex differences may play a role for the experience of PCS, their impact may be comparatively small in pre-teen children and may therefore not be detected by proxies. To further improve current clinical practice, increased efforts should be made to enable a more thorough assessment of neuropsychiatric outcomes after pTBI, including sex-specific biomarkers.

### Strengths and limitations

We conducted the first systematic investigation of the psychometric properties of the German RPQ proxy version after pTBI. The results presented highlight the applicability of the RPQ proxy as a valid tool for measuring PCS in pTBI samples. As such, the study has a number of strengths. The validation process adhered to most of the methodological standards proposed in the COSMIN checklist (i.e., internal consistency, criterion validity, structural validity, hypotheses testing) [27]. Importantly, our study demonstrated high correlations between the RPQ proxy ratings and the PCSI-P, the instrument recommended by the CDE for assessing PCS after pTBI. The recruitment process was based on specific inclusion and exclusion criteria for validation studies, e.g. [73]. , and resulted in a pTBI sample which was suitable for a thorough examination of psychometric quality. Furthermore, we have provided evidence for the construct validity of the original one-factor structure and the well-established three-factor model. An added value of the current study was the comparison of DIF between the current study population, consisting of proxy ratings for children after pTBI aged 8—12 years, and the recently published [25] data of proxy ratings for adolescents after TBI aged 13—17 years. Overall, the results of these DIF analyses show that the RPQ proxy can be used to rate PCS in both children and adolescents. Thus, the evaluation of TBI-affected populations at different ages or over the course of an individual’s recovery from a lifespan perspective is to be encouraged.

However, the current study also has some limitations. First, less than 10% of the families that received an invitation took part in the QOLIBRI-KID/ADO study. Therefore, a potential sample bias in the presented data cannot be ruled out. We observed that parents were unwilling to participate in the QOLIBRI-KID/ADO study if their child had sustained a severe pTBI with serious negative consequences because of the risk of re-traumatization, or if their child did not experience any symptoms after pTBI, because the parents felt that participation would not be beneficial either to the study or to their child. The resulting lack of variance in pTBI severity may somewhat limit our findings on the psychometric properties of the RPQ proxy version. Second, the study population was relatively small and heterogeneous with regard to sociodemographic (e.g., gender) and injury-related (e.g., TBI severity, number of lesions) characteristics. Nonetheless, a priori sample size estimations suggested that our study had sufficient power to detect relevant effects, allowing us to draw robust conclusions about the psychometric quality of the RPQ proxy after pTBI. The effect of potential factors influencing the RPQ scores (e.g., functional recovery, time since injury) remains to be studied using larger pTBI samples. Moreover, construct validity analyses revealed correlations between the PHQ-9 and GAD-7 scores and the cognitive and somatic scales of the RPQ proxy. Future studies might focus on different candidate constructs to assess divergent validity, such as communication behavior [74]. The RPQ proxy ratings were most frequently provided by mothers (76%), which is commonly the case for health-related proxy ratings [75]. To date, gender effects in proxy ratings have rarely been investigated, with one experimental study reporting that fathers tended to more accurate judges of children’s pain experience than mothers [76]. Further research should therefore assess the accuracy of proxy ratings compared with self-reports for assessing PCS, particularly after pTBI, and investigate the determinants of accurate assessments. Moreover, most children (82%) in the current sample had experienced a pTBI between two and ten years before study enrollment. Consequently, the validity of RPQ proxy ratings in acute phases after TBI remains to be examined in more depth. In addition, the current study was not able to provide further validation of the previously proposed cut-off values for clinically relevant RPQ scores in the field of pTBI [35]. Further research is therefore needed to provide robust conclusions on how to capture symptom burden after pTBI. Finally, as discussed above, although the CFA results supported the three-factor model based on the goodness-of-fit indices, further external validation is needed. A recent study by our group [18] provides evidence for the sensitivity of the RPQ, among other outcome measures, across sociodemographic (i.e., sex, age, education), premorbid (psychological health status), and injury-related (i.e., clinical care pathways, TBI and extracranial injury severity) factors in individuals after TBI. Replicating the three-factor model using additional samples, including specifications for different group characteristics (i.e., multi-group CFA), would provide further support for the stability of the RPQ proxy scoring.

Future research should aim to establish reference values for a general, brain-healthy population for the RPQ proxy in German as well as in other languages in order to allow a better assessment of the clinical relevance of symptom burden after pTBI [77]. Since the RPQ has demonstrated clinical as well as research-related applicability, the collection of reference values from healthy samples will enable clinicians to identify individuals who are severely impacted after TBI as targets for clinical treatment. These reference values will also support research into distinct predictors of symptom burden after TBI. Consequently, the relationship between RPQ proxy ratings and psychosocial and injury-related factors should be studied in more detail. Candidate predictors of somatic, cognitive, and emotional PCS (e.g., age, gender, education) have been identified in adults [78] and could play an integral role in the therapeutic and rehabilitative process. Finally, systematic investigations of RPQ self-reports after pTBI are scarce. Subjective ratings of PCS should be preferred when assessing the reliability of the RPQ proxy and can provide a useful source of information for clinical practice.

## Conclusions

The current study is the first to present a systematic psychometric evaluation of the German RPQ proxy version in children following pTBI. Our findings indicate good to excellent psychometric properties for this instrument. Moreover, we have provided evidence for the comparability of RPQ proxy ratings in children and in adolescents. The current work therefore adds to previous findings on the validity of the RPQ in adolescents [25] and adults [14] and underlines the clinical utility of the German RPQ for assessing PCS across the lifespan of individuals after TBI. Future research should further investigate the validity of RPQ proxy assessments in younger children (i.e., < 8 years) and of RPQ self-reports in children aged 8—12 years. Moreover, RPQ proxy ratings should more often be interpreted in the context of parental well-being. An increase in the use of RPQ self-reports and proxy assessments for cognitive, somatic, and emotional PCS may inform clinicians about individuals in need of personalized treatment (e.g., neuropsychological trainings, physical therapy, psychological counselling).

### Electronic supplementary material

Below is the link to the electronic supplementary material.

Supplementary Material 1

## Data Availability

The datasets used and/or analyzed during the current study are available from the corresponding author on reasonable request.

## Abbreviations

CDE: Common Data Elements
CFA: Confirmatory Factor Analysis
CFI: Comparative Fit Index
CITC: Corrected Item-Total Correlation
DIF: Differential Item Functioning Analysis
GAD-7: Generalized Anxiety Disorder Scale 7
GCS: Glasgow Coma Scale
KOSCHI: Kings Outcome Scale for Childhood Head Injury
LORDIF: Logistic Ordinal Regression
PCS: Post-Concussion Symptoms
PCSI-P: Post-Concussion Symptoms Inventory Proxy Version
PHQ-9: Patient Health Questionnaire 9
PROM: Patient-Reported Outcome Measure
pTBI: Pediatric Traumatic Brain Injury
QOLIBRI-KID/ADO: Quality of Life after Brain Injury for Children and Adolescents study
RMSEA: Root Mean Square Error of Approximation
RPQ: Rivermead Post-Concussion Symptoms Questionnaire
SRMR: Standardized Root Mean Square Residual
TLI: Tucker-Lewis-Index
WLSMV: Weighted Least Squares Estimator
