# Supplementary material for: Applicability and clinical utility of the German rivermead post-concussion symptoms questionnaire in proxies of children after traumatic brain injury: an instrument validation study
Source: BMC Neurol. 2024 Apr 19;24:133. doi: 10.1186/s12883-024-03587-2 (PMC11027521; doi:10.1186/s12883-024-03587-2)
Supplement: Supplementary file 1 — Supplementary Material 1 [file 12883_2024_3587_MOESM1_ESM.docx]

Applicability and Clinical Utility of the German Rivermead Post-Concussion Symptoms Questionnaire in Proxies of Children after Traumatic Brain Injury: An Instrument Validation Study ­­— Supplementary Material

Fabian Bockhop, Sven Greving, Marina Zeldovich, Ugne Krenz, Katrin Cunitz, Dagmar Timmermann, Matthias Kieslich, Nada Andelic, Anna Buchheim, Inga K. Koerte, Maike Roediger, Knut Brockmann, Michaela V. Bonfert, Steffen Berweck, Michael Lendt, Michael Staebler, and Nicole von Steinbuechel

**Table S1. Descriptive statistics for proxy-reported RPQ items (N = 152).**

| Symptom | Factor | M | SD | SK | KU | Floor | Ceiling | % Missing |
| --- | --- | --- | --- | --- | --- | --- | --- | --- |
| Headaches | S | 0.88 | 1.10 | 1.23 | 0.76 | 47 | 4 | 3.95 |
| Feeling of Dizziness | S | 0.25 | 0.64 | 3.18 | 11.52 | 78 | 1 | 5.26 |
| Nausea and/or Vomiting | S | 0.25 | 0.70 | 3.56 | 13.53 | 80 | 1 | 4.61 |
| Noise Sensitivity, easily upset by loud Noise | S | 0.77 | 1.07 | 1.33 | 0.91 | 53 | 3 | 3.95 |
| Sleep Disturbance | S | 0.71 | 1.07 | 1.65 | 2.03 | 55 | 4 | 4.61 |
| Fatigue, tiring more easily | S | 0.62 | 0.93 | 1.46 | 1.32 | 59 | 1 | 3.95 |
| Being Irritable, easily angered | E | 1.13 | 1.16 | 0.87 | -0.13 | 35 | 5 | 4.61 |
| Feeling Depressed or Tearful | E | 0.55 | 0.90 | 1.67 | 2.05 | 63 | 1 | 3.95 |
| Feeling Frustrated or Impatient | E | 1.10 | 1.18 | 0.88 | -0.29 | 38 | 4 | 3.95 |
| Forgetfulness, poor memory | C | 0.73 | 0.98 | 1.24 | 0.57 | 53 | 1 | 3.95 |
| Poor Concentration | C | 1.00 | 1.13 | 0.82 | -0.58 | 43 | 1 | 3.95 |
| Taking Longer to Think | C | 0.57 | 0.93 | 1.67 | 1.97 | 61 | 1 | 4.61 |
| Blurred Vision | S | 0.29 | 0.75 | 2.95 | 8.94 | 80 | 1 | 4.61 |
| Light Sensitivity, easily upset by bright light | S | 0.36 | 0.84 | 2.46 | 5.61 | 77 | 1 | 3.95 |
| Double Vision | S | 0.10 | 0.38 | 4.07 | 16.10 | 89 | 0 | 3.95 |
| Restlessness | E | 0.51 | 0.90 | 1.81 | 2.47 | 66 | 1 | 3.95 |

Note. *M* = mean, *SD* = standard deviation, *KU* = kurtosis, *SK* = skewness, S = somatic scale, E = emotional scale, C = cognitive scale. Floor and ceiling indicate the percentage of responses in the lowest (i.e., 0 = ‘not experienced at all’) and the highest (i.e., 4 = ‘severe problem’) two response categories, respectively.

**Table S2. Comparisons of proxy-assessed RPQ total and scale scores concerning sociodemographic and injury‑related characteristics.**

| Variable | Scale | Group | *n* | *M* | *Mdn* | *Cliff’s δ* | *U* | *p* |
| --- | --- | --- | --- | --- | --- | --- | --- | --- |
| Sex | Total | Male | 89 | 6.13 | 2 | 0 | 2544.5 | 0.514 |
|  |  | Female | 57 | 7.18 | 2 |  |  |  |
|  | Cognitive | Male | 89 | 1.44 | 0 | -0.04 | 2441.5 | 0.319* |
|  |  | Female | 57 | 1.7 | 0 |  |  |  |
|  | Emotional | Male | 89 | 2.19 | 0 | -0.02 | 2478 | 0.394* |
|  |  | Female | 57 | 2.33 | 0 |  |  |  |
|  | Somatic | Male | 89 | 2.51 | 0 | -0.05 | 2413 | 0.295* |
|  |  | Female | 57 | 3.14 | 0 |  |  |  |
| TBI  severity | Total | Mild | 103 | 4.81 | 0 | -0.35 | 1430.5 | **<0.001** |
|  |  | Moderate—Severe | 43 | 10.7 | 6 |  |  |  |
|  | Cognitive | Mild | 103 | 1.08 | 0 | -0.33 | 1481 | **<0.001*** |
|  |  | Moderate—Severe | 43 | 2.65 | 2 |  |  |  |
|  | Emotional | Mild | 103 | 1.67 | 0 | -0.29 | 1581.5 | **0.001*** |
|  |  | Moderate—Severe | 43 | 3.63 | 2 |  |  |  |
|  | Somatic | Mild | 103 | 2.06 | 0 | -0.32 | 1504.5 | **<0.001*** |
|  |  | Moderate—Severe | 43 | 4.42 | 2 |  |  |  |
| KOSCHI  score | Total | 5b | 123 | 4.26 | 0 | *-0.75* | 332.5 | **<0.001** |
|  |  | <5b | 22 | 18.82 | 20 |  |  |  |
|  | Cognitive | 5b | 123 | 0.96 | 0 | *-0.62* | 520 | **<0.001*** |
|  |  | <5b | 22 | 4.59 | 5 |  |  |  |
|  | Emotional | 5b | 123 | 1.59 | 0 | *-0.56* | 590.5 | **<0.001*** |
|  |  | <5b | 22 | 5.77 | 6 |  |  |  |
|  | Somatic | 5b | 123 | 1.72 | 0 | *-0.69* | 421.5 | **<0.001*** |
|  |  | <5b | 22 | 8.45 | 5 |  |  |  |
| Post-TBI sensory, cognitive, and/or physical problems | Total | No problems | 96 | 3.12 | 0 | -0.56 | 1061.5 | **<0.001** |
|  |  | At least one problem | 50 | 13.1 | 11.5 | -0.56 | 1061.5 |  |
|  | Cognitive | No problems | 96 | 0.5 | 0 | -0.52 | 1144 | **<0.001*** |
|  |  | At least one problem | 50 | 3.54 | 2.5 | -0.52 | 1144 |  |
|  | Emotional | No problems | 96 | 1.26 | 0 | -0.44 | 1353 | **<0.001*** |
|  |  | At least one problem | 50 | 4.14 | 4 | -0.44 | 1353 |  |
|  | Somatic | No problems | 96 | 1.36 | 0 | -0.49 | 1212.5 | **<0.001*** |
|  |  | At least one problem | 50 | 5.42 | 4 | -0.49 | 1212.5 |  |
| PHQ-9 | Total | < 5 | 98 | 2.5 | 0 | *-0.71* | 685.5 | **<0.001** |
|  |  | ≥ 5 | 48 | 14.79 | 13 |  |  |  |
|  | Cognitive | < 5 | 98 | 0.5 | 0 | *-0.51* | 1152.5 | **<0.001*** |
|  |  | ≥ 5 | 48 | 3.67 | 3 |  |  |  |
|  | Emotional | < 5 | 98 | 0.94 | 0 | *-0.59* | 963.5 | **<0.001*** |
|  |  | ≥ 5 | 48 | 4.92 | 5.5 |  |  |  |
|  | Somatic | < 5 | 98 | 1.06 | 0 | *-0.63* | 874 | **<0.001*** |
|  |  | ≥ 5 | 48 | 6.21 | 5 |  |  |  |
| GAD-7 | Total | < 5 | 97 | 2.6 | 0 | *-0.7* | 703.5 | **<0.001** |
|  |  | ≥ 5 | 49 | 14.35 | 12 |  |  |  |
|  | Cognitive | < 5 | 97 | 0.65 | 0 | *-0.45* | 1317.5 | **<0.001*** |
|  |  | ≥ 5 | 49 | 3.31 | 2 |  |  |  |
|  | Emotional | < 5 | 97 | 0.67 | 0 | *-0.7* | 715.5 | **<0.001*** |
|  |  | ≥ 5 | 49 | 5.37 | 6 |  |  |  |
|  | Somatic | < 5 | 97 | 1.28 | 0 | *-0.56* | 1034 | **<0.001*** |
|  |  | ≥ 5 | 49 | 5.67 | 4 |  |  |  |

* Bonferroni‑adjusted significance level for scale comparisons was 5%/3 = 1.67%.

Note. TBI = traumatic brain injury, KOSCHI = Kings Outcome Scale for Childhood Head Injury, Post-TBI sensory, cognitive, and/or physical problems = parent-reported post-TBI problems, PHQ‑9 = Patient Health Questionnaire 9, GAD‑7 = Generalized Anxiety Disorder Scale 7, *n* = sample size, *M* = mean, *Mdn* = median, *U* = Mann-Whitney U‑test statistic, *p* = p‑value in difference test, Cliff’s δ = effect statistic: δ < |0.28| (small), |0.28| < δ < |0.43| (medium), and δ ≥ |0.43| (large). Values in *italics* indicate large effects. Negative values indicate greater impairment in individuals with less favorable recovery or more severely rated symptoms. p‑values in **bold** indicate significant results (either on 5% or on 1.67% α level).

**Table S3. Summary of Differential Item Functioning Analyses.**

| Symptom | Factor | *p* | McFadden *R*² |
| --- | --- | --- | --- |
| Headaches | S | 0.104 |  |
| Feeling of Dizziness | S | 0.085 |  |
| Nausea and/or Vomiting | S | 0.610 |  |
| Noise Sensitivity, Easily Upset by Loud Noise | S | 0.033 |  |
| Sleep Disturbance | S | 0.42 |  |
| Fatigue, Tiring More Easily | S | 0.066 |  |
| Being Irritable, Easily Angered | E | 0.573 |  |
| Feeling Depressed or Tearful | E | 0.301 |  |
| Feeling Frustrated or Impatient | E | 0.206 |  |
| Forgetfulness, Poor Memory | C | 0.007* | 0.013 |
| Poor Concentration | C | 0.203 |  |
| Taking Longer to Think | C | 0.027 |  |
| Blurred Vision | S | 0.714 |  |
| Light Sensitivity, Easily Upset by Bright Light | S | 0.304 |  |
| Double Vision | S | 0.465 |  |
| Restlessness | E | 0.525 |  |

* = p-value < 0.01.

Note. S = somatic scale, E = emotional scale, C = cognitive scale, p-values refer to *χ*²-tests between LORDIF models regressing with individual symptom ratings as outcome variables. The respective predictors in the regression models were (1) scale means, and (2) scale means, age category, as well as age-group-scale-mean interaction. McFadden’s R² is only reported for items with significant differences in model comparison.
